# Performance of the QIAstat-Dx Gastrointestinal Panel for Diagnosing Infectious Gastroenteritis

**DOI:** 10.1128/JCM.01737-19

**Published:** 2020-02-24

**Authors:** Stefan A. Boers, Cas J. A. Peters, Els Wessels, Willem J. G. Melchers, Eric C. J. Claas

**Affiliations:** a Department of Medical Microbiology, Leiden University Medical Center, Leiden, The Netherlands; b Department of Medical Microbiology, Radboud University Medical Center, Nijmegen, The Netherlands; Brigham and Women’s Hospital

**Keywords:** QIAstat-Dx, gastroenteritis, gastrointestinal panel, molecular diagnostics, syndromic testing

## Abstract

Detection and identification of enteropathogens that cause infectious gastroenteritis are essential steps for appropriate patient treatment and effective isolation precautions. Several syndrome-based tests have recently become available, with the gastrointestinal panel (GIP) assay on the QIAstat-Dx as the most recent addition to the syndromic testing landscape. The QIAstat-Dx GIP assay offers simultaneous testing for 24 bacterial, viral, and parasitic enteropathogens using a single test that reports the results in 70 min. In this study, we compared the performance of the GIP assay to laboratory-developed real-time PCR assays (LDTs), using 172 prospectively and retrospectively collected fecal samples from patients suspected to have infectious gastroenteritis. The GIP assay detected 97/107 enteropathogens (91%) that were detected by LDTs, and the overall agreement of results increased to 95% when excluding discrepant results with cycle threshold (*C_T_*) values of >35. Further, the GIP assay detected 42 additional enteropathogens that were not detected, or tested, by LDTs. These included 35 diarrheagenic Escherichia coli targets for which the clinical relevance is unclear for most. The main advantage of the QIAstat-Dx system compared to other syndromic testing systems is the ability to generate *C_T_* values that could help with the interpretation of results. However, compared to LDTs, the GIP assay is limited by flexibility and high-throughput testing. In conclusion, the GIP assay offers an easy, sample-to-answer workflow with a rapid detection of the most common enteropathogens and therefore has the potential to direct appropriate therapy and infection control precautions.

## Abstract (precis)

Detection and identification of enteropathogens that cause infectious gastroenteritis are essential steps for appropriate patient treatment and effective isolation precautions. Several syndrome-based tests have recently become available, with the gastrointestinal panel (GIP) assay on the QIAstat-Dx as the most recent addition to the syndromic testing landscape.

## INTRODUCTION

Infectious gastroenteritis is an inflammation of the mucosa of the stomach, small intestine, and/or large intestine caused by infections with viruses, bacteria, or parasites. It is one of the most common diseases throughout the world (1). In the Netherlands, an estimated incidence of 0.29 episode/person-year has been observed (2). Most episodes of infectious gastroenteritis are brief and self-limiting, at least in the Western world. However, persistent or severe infections can lead to hospitalization, especially in infants, the elderly, and immunocompromised patients, who have an increased risk of dehydration (2, 3). In addition, nosocomial infectious gastroenteritis is a common complication in hospitalized patients that contributes to morbidity and mortality and increases the length of stay and hospital costs (4, 5). Rapid and reliable microbiological diagnosis of infectious gastroenteritis is essential to ensure initiation of appropriate antimicrobial therapy and timely implementation of isolation precautions.

Laboratory methods for diagnosing infectious gastroenteritis have evolved over time, and PCR-based assays have now become the mainstay. For example, multiplex real-time PCR (RT-PCR) assays have been developed and implemented for routine diagnostics to detect and identify multiple pathogens in a single test with high sensitivity and specificity (6). However, the maximum number of different pathogens that can be detected simultaneously in a single multiplex RT-PCR assay is limited due to primer/probe design considerations and the fact that most PCR instruments can detect no more than four or five different fluorescently labeled probes. As a result, several multiplex RT-PCR assays need to be performed to cover the wide range of enteropathogens that can cause infectious gastroenteritis. To overcome this limitation, commercially available molecular syndromic testing systems have been developed that combine nucleic acid extraction, amplification, and detection of a wide range of targets in a single test (7, 8). These systems offer a relatively easy sample-to-answer workflow with a turnaround time of less than 2 h, making them suitable for decentralized or even point-of-care testing (POCT).

**FIG 1:**
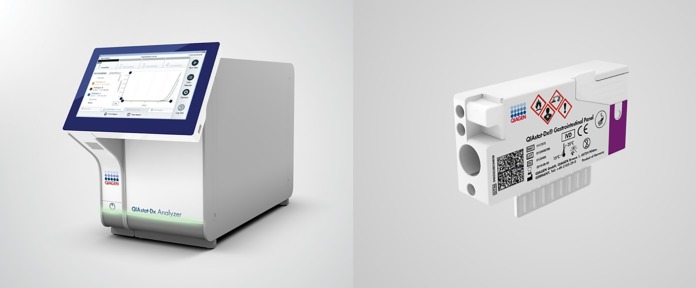
The QIAstat-Dx system (left) and corresponding cartridge of the QIAstat-Dx GIP assay (right).

Syndromic testing panels, for example, the BioFire FilmArray gastrointestinal panel (GIP) (bioMérieux) or xTAG GIP (Luminex), are based on endpoint detection of PCR products and therefore do not provide a quantitative indication (e.g., cycle threshold [*C_T_*] value) of the detected enteropathogens. Recently, Qiagen launched a novel gastrointestinal panel for the QIAstat-Dx (formerly STAT-Dx; DiagCORE) RT-PCR-based syndromic testing system, which does provide *C_T_* values. The QIAstat-Dx GIP assay enables simultaneous testing for 24 viral, bacterial, and parasite enteropathogens, including Clostridium difficile toxin A/B, enteroaggregative Escherichia coli (EAEC), enteropathogenic E. coli (EPEC), enterotoxigenic E. coli (ETEC) LT/ST, Shiga-like-toxin-producing E. coli (STEC) *stx*_1_/*stx*_2_, STEC O157:H7, enteroinvasive E. coli (EIEC)/*Shigella*, pathogenic *Campylobacter* spp. (Campylobacter jejuni, C. upsaliensis, and C. coli), Plesiomonas shigelloides, *Salmonella* spp., Vibrio cholerae, Vibrio parahaemolyticus, Vibrio vulnificus, Yersinia enterocolitica, Cyclospora cayetanensis, *Cryptosporidium* spp., Entamoeba histolytica, Giardia lamblia, adenovirus F40/41, astrovirus, norovirus GI, norovirus GII, rotavirus, and sapovirus (I, II, IV, and V). All of the sample preparation and analysis steps are performed automatically within disposable plastic cartridges and can be completed in 70 min (Fig. 1).

In this study, we compared the performance of the QIAstat-Dx GIP assay with those of laboratory-developed (multiplex) RT-PCR assays (LDTs) that are used as a routine diagnostic tool in the university medical centers of Leiden (LUMC-Leiden) and Nijmegen (RUMC-Nijmegen) in the Netherlands. Performance was tested using remnant anonymized fecal samples that had been submitted for the diagnosis of infectious gastroenteritis.

## MATERIALS AND METHODS

### Clinical samples.

A total of 172 fecal samples from 170 patients were included in this study that consisted of a retrospective panel of 95 samples and a prospective panel of 77 samples. All 95 retrospective samples had previously been submitted and tested prospectively for diagnosis of infectious gastroenteritis at either the Leiden University Medical Center (LUMC-Leiden) or the Radboud University Medical Center Nijmegen (RUMC-Nijmegen). Aliquots of these samples were stored at −80°C and were available for use in retrospective analysis. For this, a selection of samples was made using the laboratory information management systems to include most GIP targets. In addition, from December 2018, 77 fecal samples from patients with suspected infectious gastroenteritis were subjected randomly to prospective diagnostic screening by LDTs and GIP testing at both institutes. All samples included in this study have been anonymized and are not traceable to individual patients, omitting the need for approval by an ethical committee.

### LDT comparator testing.

Briefly, nucleic acids were extracted from 200 μl of bead-beaten fecal samples and eluted in 50 μl or 100 μl elution buffer at the RUMC-Nijmegen and the LUMC-Leiden, respectively, using a MagNA Pure 96 instrument (Roche). Five-microliter and 10-μl volumes of the nucleic acid extracts at the RUMC-Nijmegen and the LUMC-Leiden, respectively, were tested in monoplex or multiplex LDTs, designed to detect a variety of bacterial, parasitic, and viral pathogens that can cause infectious gastroenteritis, with updated versions (if necessary) of primers and probes as previously described (see Table S1 in the supplemental material) (9–26). Amplification and detection were performed on a LightCycler 480 PCR instrument (Roche) and a Bio-Rad CFX96 real-time PCR instrument (Bio-Rad) at the RUMC-Nijmegen and the LUMC-Leiden, respectively. All LDTs have been implemented for routine diagnostic use after validation according to the ISO 15189:2012 guideline for clinical laboratories.

### QIAstat-Dx GIP testing.

The GIP assay was performed as described in the manufacturer’s instructions. In short, approximately 25 to 100 mg of unpreserved thawed feces (retrospective panel) or fresh feces (prospective panel) was resuspended in 1 ml of Cary-Blair transport medium. After vortexing, 200 μl of the resuspended sample was inserted into a GIP test cartridge, which contains all the reagents necessary to isolate and amplify nucleic acids from the resuspended sample. The barcode of the GIP test cartridge and the barcode of the corresponding sample were scanned by the QIAstat-Dx operational module followed by loading the GIP test cartridge into the QIAstat-Dx analyzer module and starting the run. The detected real-time amplification signals were automatically interpreted by the integrated software and reported to the user by the QIAstat-Dx operational module. Each report contains the results obtained for all 24 viral, bacterial, and parasite targets, as well as for the internal control (IC). The IC verifies all steps of the analysis process, including homogenization of samples, lysis of viral and cellular structures, nucleic acid purification, reverse transcription, and real-time PCR. A positive signal for the IC—regardless of the *C_T_* value—produces a valid GIP assay result, while a negative signal from the IC invalidates all negative results in the analysis (excluding the detected and identified GIP assay targets).

### Discrepant analysis.

In the case of discrepant results, the discordant sample was retested with a new GIP test cartridge if the LDT tested positive and the GIP test was negative (LDT^+^/GIP^−^) or with the LDT in the case of LDT^−^/GIP^+^ results. Each discordant sample with sufficient volume available and that was not resolved by the first round of discrepant analysis was retested using the LDTs by the other laboratory.

## RESULTS

A total of 172 fecal samples from 170 patients were included in this study that consisted of 95 retrospective and 77 prospective samples. Ninety-seven of these 172 samples tested positive for at least one enteropathogen using LDTs, and no enteropathogen was detected in the remaining 75 samples (Table 1). For 154/172 samples (90%), GIP testing was completed at the first attempt. Of the 18 samples with an initial invalid result, 12 samples generated a valid GIP assay result and 6 samples failed again upon retesting.

**TABLE 1:** Number of fecal samples included in this study^*a*^

Institute	No. of samples				Total
	Retrospective samples		Prospective samples		
	LDT positive	LDT negative	LDT positive	LDT negative	
LUMC-Leiden	43	9	16	29	97
RUMC-Nijmegen	33	10	5	27	75
Total	76	19	21	56	172

a LDT, laboratory-developed (multiplex) RT-PCR assays; LUMC, Leiden University Medical Center; RUMC, Radboud University Medical Center.

The performance characteristics for individual GIP targets are presented in Table 2. Concordance with LDTs was 100% for STEC *stx*_1_/*stx*_2_ (5/5), EIEC/*Shigella* (6/6), pathogenic *Campylobacter* spp. (9/9), *Salmonella* spp. (7/7), adenovirus F40/41 (3/3), astrovirus (5/5), norovirus GI (6/6), norovirus GII (10/10), rotavirus (6/6), and sapovirus (4/4) and 93% for C. difficile toxin A/B (13/14). For other targets, for which only a limited number of positives had been tested, no complete concordance was observed: for P. shigelloides 4/5 positives were detected in comparison to the LDT, for Y. enterocolitica 2/5, *C. cayetanensis* 2/3, *Cryptosporidium* spp. 6/7, E. histolytica 2/4, and *G. lamblia* 7/8. The overall agreement for the GIP assay with targets detected by LDTs was shown to be 97 of the 107 enteropathogens (91%). The detection of enteropathogens in samples containing a single enteropathogen was concordant in 76/82 (92.7%) samples. For samples containing multiple enteropathogens, the same enteropathogens as were detected by LDTs could also be identified by the GIP assay in 8/9, 1/1, and 0/1 in the case of two, three, and four enteropathogens present, respectively.

**TABLE 2:** Comparison of rates of enteropathogen detection by LDTs and the GIP assay

QIAstat-Dx GIP target	No. of results			
	LDT^+^/GIP^+^	LDT^+^/GIP^−^	LDT^−^/GIP^+^	LDT^NP^^*c*^/GIP^+^
Bacterial				
Clostridium difficile toxin A/B	13	1		
EAEC^*a*^				13
EPEC^*a*^				17
ETEC LT/ST^*a*^				4
STEC *stx*_1_/*stx*_2_	5			
STEC O157:H7^*a*^				1
EIEC^*d*^/*Shigella*	6			
Pathogenic *Campylobacter* spp.	9		3	
Plesiomonas shigelloides	4	1		1^*a*^
*Salmonella* spp.	7			
Vibrio cholerae^*a*^				
Vibrio parahaemolyticus^*a*^				
Vibrio vulnificus^*a*^				
Yersinia enterocolitica	2	3		
Parasites				
Cyclospora cayetanensis	2	1		
*Cryptosporidium* spp.	6	1	1	
Entamoeba histolytica	2	2		
Giardia lamblia	7	1		
Viruses				
Adenovirus F40/41	3			
Astrovirus	5		1	
Norovirus GI + norovirus GII^*b*^	16		1	
Rotavirus	6			
Sapovirus (I, II, IV, V)	4			
Total	97	10	6	36

a Detection of enteroaggregative E. coli (EAEC), enteropathogenic E. coli (EPEC), enterotoxigenic E. coli (ETEC), Shiga-like-toxin-producing E. coli (STEC) O157:H7, and *Vibrio* spp. are not part of the routine diagnostics at both institutes and is therefore not tested using LDTs. In addition, RUMC-Nijmegen does not use a LDT for the detection of P. shigelloides.

b The LDT performed at RUMC-Nijmegen does not differentiate between norovirus GI and norovirus GII. In total, six norovirus GI and 11 norovirus GII were detected using GIP testing.

c NP, not performed.

d EIEC, enteroinvasive E. coli.

**FIG 2:**
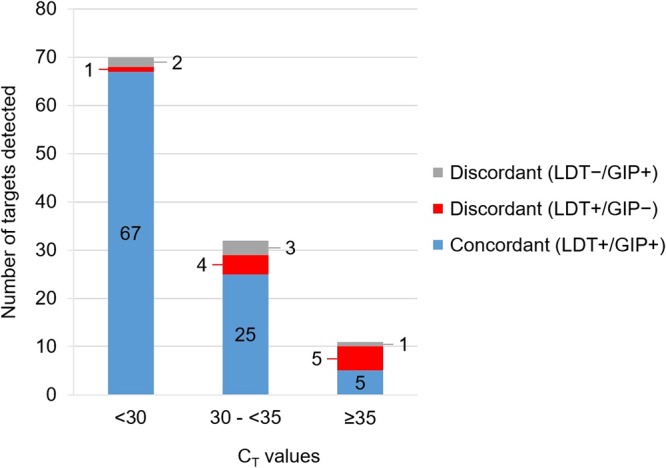
Comparison of rates of enteropathogen detection by LDT and the GIP assay by *C_T_* value.

As shown in Fig. 2, a total of 10 discordant results (LDT^+^/GIP^−^) were obtained, with only one target with a *C_T_* value of less than 30 (i.e., *Cryptosporidium* spp.), four targets with *C_T_* values between 30 and 35 (i.e., Y. enterocolitica, *C. cayetanensis*, and two E. histolytica results), and five targets with *C_T_* values of 35 or higher (i.e., C. difficile toxin A/B, P. shigelloides, *G. lamblia*, and two Y. enterocolitica results). Although all discrepant results with *C_T_* values lower than 35 were confirmed by repeat testing using both LDTs and the GIP assay, none of these samples contained enough sample volume for further characterization using comparator methods and so the discrepancy could not be resolved (Table 3). Excluding LDT^+^/GIP^−^ discrepant results with *C_T_* values of 35 or higher, considered to be of questionable clinical relevance anyway, increased the overall agreement of results to 95%.

**TABLE 3:** Discrepant analysis results^*a*^

No.	LDT result (*C_T_* value)	GIP result (*C_T_* value)	Discrepant testing result (*C_T_* value)
Fecal samples initially tested at LUMC-Leiden			
1	No pathogens detected	***Cryptosporidium* spp.** (34.1)	NP^*b*^
2	**C. difficile** (38.0)	None	NP^*b*^
3	**P. shigelloides** (37.0), norovirus GII (20.7)	Norovirus GII (23.5), ETEC (15.2), EAEC (18.7)	NP^*b*^
4	**Y. enterocolitica** (32.4)	EPEC (28.6)	NP^*b*^
5	**Y. enterocolitica** (37.2)	No pathogens detected	NP^*c*^
6	***C. cayetanensis*** (31.9)	EPEC (21.3)	NP^*b*^
7	*Cryptosporidium* spp. (27.2), *Campylobacter* spp. (33.3), ***G. liamblia*** (35.0), **E. histolytica** (33.2)	*Cryptosporidium* spp. (18.6), *Campylobacter* spp. (33.4)	NP^*b*^
8	**Y. enterocolitica** (37.4)	No pathogens detected	NP^*c*^
9	**E. histolytica** (33.5)	No pathogens detected	NP^*b*^
Fecal samples initially tested at RUMC-Nijmegen			
10	No pathogens detected	**Astrovirus** (37.9)	NP^*c*^
11	*G. lamblia* (30.0)	*G. lamblia* (28.5), ***Campylobacter* spp.** (33.9)	*Campylobacter* spp. (41.0)^*c*^
12	***Cryptosporidium* spp.** (26.0)	No pathogens detected	NP^*b*^
13	C. difficile (28.2)	C. difficile (27.9), ***Campylobacter* spp.** (20.5), ETEC (18.0)	Campylobacter coli (26.1)
14	No pathogens detected	***Campylobacter* spp.** (25.2)	Campylobacter jejuni (26.0)
15	No pathogens detected	**Norovirus GII** (31.4)	Norovirus GII (21.4)

a All discrepant results with *C_T_* values lower than 35 were confirmed by repeated testing using both LDTs and the GIP assay, for which only the latest test results are shown. Enteropathogens in bold were detected by only one of the two methods used, either LDTs or the GIP assay. Discrepant testing was performed using the LDTs of the laboratory that did not initially select and test the samples. The additional detection of EAEC, EPEC, ETEC, and STEC O157:H7 by the GIP assay performed at both institutes, and the additional detection of P. shigelloides by the GIP assay performed at RUMC-Nijmegen, were not included for discrepant testing because no LDTs were available to detect these targets in routine LDT testing.

b Discrepant analysis was not performed because there was not enough sample volume available.

c Discrepant analysis was not performed because the discrepant result was detected with a *C_T_* value of 35 or higher.

The GIP assay detected six additional enteropathogens in six samples that were not detected by LDTs (LDT^−^/GIP^+^). These included two targets with *C_T_* values lower than 30 (i.e., two pathogenic *Campylobacter* spp.), three targets with *C_T_* values between 30 and 35 (i.e., *Cryptosporidium* spp., pathogenic *Campylobacter* spp., and norovirus GII), and one target with a *C_T_* value of >35 (i.e., astrovirus). Again, all discrepant results with *C_T_* values lower than 35 were confirmed by repeat testing using both methods. In addition, 4/6 discrepant results obtained at the RUMC-Nijmegen were resolved using the LDTs of the LUMC-Leiden, which confirmed the detection of pathogenic *Campylobacter* spp. in three samples and norovirus GII in one sample. The remaining discrepant result with a *C_T_* value lower than 35 (i.e., *Cryptosporidium* spp.) could not be resolved because there was not enough sample volume available for additional tests (Table 3). Further, GIP testing resulted in the detection of 36 additional targets in samples for which no LDTs were available that could detect these targets at the corresponding institute. These included 17 EPEC, 13 EAEC, 4 ETEC, 1 STEC O157:H7 (that was detected as STEC by LDT), and 1 P. shigelloides result. The P. shigelloides result could be confirmed by the BioFire FilmArray gastrointestinal panel assay. As could be expected (27), most of the diarrheagenic E. coli pathotypes (i.e., EPEC [10/17], EAEC [13/13], ETEC [4/4], EIEC/*Shigella* [2/6], and STEC [3/5]) were detected by the GIP assay in samples containing two or more enteropathogens that indicated coinfection or colonization.

**FIG 3:**
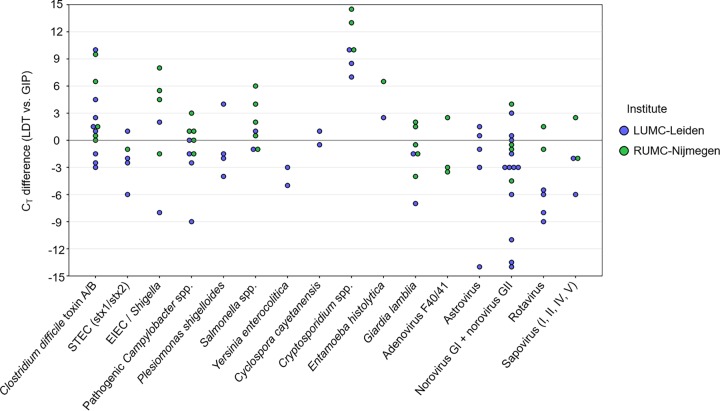
Comparison of *C_T_* values obtained with LDT and GIP assays. The *C_T_* differences are visualized by subtracting the *C_T_* values obtained by the GIP assay from the *C_T_* values obtained by LDTs for all 97 concordant (LDT^+^/GIP^+^) results. Positive values represent targets with higher *C_T_* values obtained with LDTs, whereas negative values represent targets with lower *C_T_* values obtained with LDTs than with the GIP assay.

The GIP assay reports *C_T_* values and endpoint fluorescence for each of the targets detected and the internal control (IC) used. The enteropathogen *C_T_* values obtained with the GIP assay have been compared to the corresponding enteropathogen *C_T_* values obtained with the LDT for all 97 concordant (LDT^+^/GIP^+^) results. As shown in Fig. 3, a median *C_T_* difference of 2.6 ± 3.6 has been measured between both methods, with the GIP assay resulting in lower *C_T_* values for 44 of the 97 targets (45%). Specifically, lower *C_T_* values for all *Cryptosporidium* spp. (6/6) and E. histolytica (2/2) LDT^+^/GIP^+^ results, and higher *C_T_* values for all Y. enterocolitica (2/2) LDT^+^/GIP^+^ results, were obtained using the GIP assay than with LDTs.

## DISCUSSION

In this study, we evaluated the performance, advantages, and drawbacks of the GIP assay on the QIAstat-Dx platform to detect and identify causative agents of infectious gastroenteritis. A previous study, coauthored by employees of the company, compared the performance of this GIP assay to the BioFire FilmArray gastrointestinal panel assay and demonstrated a high level of concordance for the 385 fecal samples tested (28). In our independent study, 172 fecal samples from patients with suspected infectious gastroenteritis have been tested by both the QIAstat-Dx GIP assay and validated LDTs as a comparator. The results obtained from the two laboratories showed 95% agreement (92/97) for enteropathogens detected by LDTs with *C_T_* values below 35. Unfortunately, the five discordant results (Y. enterocolitica, *Cryptosporidium* spp., *C. cayetanensis*, and two E. histolytica results) could not be resolved because insufficient sample volume limited further characterization using comparator methods. However, both fecal samples with E. histolytica LDT^+^/GIP^−^ results were part of a larger set of eight fecal samples that were obtained over a 4-month period from the same patient that all tested positive for E. histolytica with LDT, so they can be considered true positives. Although the sensitivity versus the BioFire FilmArray gastrointestinal panel assay tested 100% for E. histolytica (19/19) in the previous study (28), our data indicate a reduced limit of detection for E. histolytica by the GIP assay in comparison to our LDT. The performance of the GIP assay to detect *Vibrio* species could not be assessed due to a lack of positive fecal samples. Obviously, the performance of the GIP assay has only been evaluated on circulating strains during sample collection, meaning that a full evaluation of different genotypes of genetically diverse viruses, as for example noroviruses (29), is lacking.

The GIP assay identified six enteropathogens in six fecal samples that were not detected by LDTs (LDT^−^/GIP^+^). Five of these additional enteropathogens were detected with *C_T_* values lower than 35. Four of those were shown to be true positives, as they could be confirmed by the LDT of the LUMC-Leiden. The fifth sample tested positive for *Cryptosporidium* spp. with a *C_T_* value of 34.1. Unfortunately, there was insufficient sample volume available to perform further discrepant analysis. Potentially the enteropathogen could have been a *Cryptosporidium* species not detected by our LDTs. Analysis of our assays revealed suboptimal detection of Cryptosporidium felis, C. canis, C. meleagridis, and C. muris, which can be human enteropathogens (unpublished data). Furthermore, the GIP assay identified 45 diarrheagenic E. coli pathotypes in 36 of the 172 (21%) samples tested in this study. Concordance with LDTs was 100% for STEC and EIEC/*Shigella*, pathotypes for which the clinical relevance in disease has been firmly established (27). However, the other E. coli pathotypes are not part of the routine diagnostic portfolio of both participating institutes. Despite the fact that other syndromic gastrointestinal panels, such as those available for the BioFire FilmArray and the Seegene Allplex, also include the other E. coli pathotypes, the pathogenicity and clinical relevance of the EAEC, EPEC, and EIEC/*Shigella* pathotypes remain unclear. The majority (32/46, i.e., 70%) of the E. coli pathotypes detected were detected as coinfection or colonization with at least one (other) established enteropathogen, which is consistent with results from previous studies (28, 30, 31) and which complicates studies toward the clinical relevance and pathogenicity of E. coli pathotypes. Accurate detection methods, such as the syndromic gastrointestinal panel assays, can play an important role here.

The QIAstat-Dx GIP assay is an easy-to-perform assay that only requires the addition of an aliquot of feces in Cary-Blair transport medium into a test cartridge by a single pipetting step and reports results in 70 min. Compared to LDTs, this means a significant reduction in turnaround time, employee time and training, and potentially costs. Previous studies have shown health care cost reduction by implementing molecular syndromic testing, as it resulted in a decrease in the number of days that patients were kept in isolation and a reduced overall hospital length of stay (32, 33). The advantages of the gastrointestinal syndromic panel assays over LDTs are at the expense of flexibility and high-throughput testing. Molecular syndromic testing systems do not offer flexible configurations for customized testing, and in this case, only one test cartridge can be processed simultaneously per QIAstat-Dx analyzer module, with one to four QIAstat-Dx analyzer modules linked to one QIAstat-Dx operational module.

An important advantage of the QIAstat-Dx platform over comparable molecular syndromic testing systems is the ability to generate *C_T_* values for the targets detected and the IC used. The *C_T_* values of enteropathogens reported by the GIP assay provide an indication of the pathogen load, which can be helpful with the interpretation of results. It is important to note that the *C_T_* value differences obtained with both methods as presented in this study (Fig. 3) can be explained by both assay-specific factors (e.g., nucleotide extraction and PCR amplification efficiency) and sample-specific factors (e.g., nucleotide degradation). In addition, the GIP assay requires the fecal samples to be suspended in Cary-Blair transport medium, while for our LDT, the fecal sample is suspended in lysis buffer, so comparison of *C_T_* values should be interpreted with care.

The *C_T_* value of the IC is an important test parameter that provides information on potential inhibition within the examined samples (28, 34). However, interpretation of the IC by the QIAstat-Dx platform is performed qualitatively, i.e., positive (valid) or negative (invalid), and does not take into account the actual IC *C_T_* values to validate the GIP assay results. The range of *C_T_* values of the IC was from 27 to 37, indicating a >1,000-fold reduction in amplification efficacy, which might affect the GIP assay results. Therefore, it could be considered to set a threshold for the *C_T_* value of the IC, as high IC *C_T_* values could lead to underestimation of the detected enteropathogen(s), or worse, a false-negative result. Although this might lead to an increase in retesting samples and thus costs, the reliability of the GIP assay results will be improved.

In summary, the GIP assay on the QIAstat-Dx shows a good performance in comparison to the LDTs for diagnosis of infectious gastroenteritis. The significantly shorter time to results allows the clinician to guide effective patient treatment and care. Therefore, the QIAstat-Dx GIP assay has the potential to be cost-effective in relation to LDTs and can be used as a rapid syndromic testing system in many different settings.

## Supplementary Material

Supplemental file 1
