# Supplementary material for: Performance of the QIAstat-Dx Gastrointestinal Panel for Diagnosing Infectious Gastroenteritis
Source: J Clin Microbiol. 2020 Feb 24;58(3):e01737-19. doi: 10.1128/JCM.01737-19 (PMC7041566; doi:10.1128/JCM.01737-19)
Supplement: Supplemental file 1 [file JCM.01737-19-s0001.pdf]

**TABLE S1** Primers and probes used in this study for LDT comparator testing.

| Pathogen | LUMC-Leiden | | | RUMC-Nijmegen | | |
|---|---|---|---|---|---|---|
| | Target | Ref. | Nucleotide sequence (5' – 3') | Target | Ref. | Nucleotide sequence (5' – 3') |
| Adenovirus F40/41 | Hexon gene | (1) | F: CTCGACATGACTTTTGAGGT | Fiber gene | (9) | F: AACTTTCTCTCTTAATAGACGCC |
| | | | R: GTAGACGGCCTCGATGAC | | | R: AGGGGGCTAGAAAACAAAA |
| | | | **P: FAM-AGCCCACACTTCT-BHQ1**[B] | | | P: LC670-CTGACACGGGCACTCTTCGC-BBQ |
| Astrovirus | ORF1a | (1) | F: TCTYATAGACCGYATTATTGG | ORF1a | (1) | F: TCTYATAGACCGYATTATTGG |
| | | | R: TCAAATTCTACATCATCACCAA | | | R: TCAAATTCTACATCATCACCAA |
| | | | P: FAM-CCCCADCCATCATCATCTTCATCA-BHQ1 | | | P: YAK-CCCCADCCATCATCATCTTCATCA-BBQ |
| *Campylobacter coli* | *cueE* | (2) | F: AAGCTCTTATTGTTCTAACCAATTCTAACA | *cueE* | (2) | F: AAGCTCTTATTGTTCTAACCAATTCTAACA |
| | | | R: TCATCCACAGCATTGATTCCTAA | | | R: TCATCCACAGCATTGATTCCTAA |
| | | | **P: ATTO700-TTGGACCTCAATCTCGCTT-MGB-NFQ** | | | **P: YAK-TTGGACCTCAATCTCGCTT-BBQ** |
| *Campylobacter jejuni* | *mapA* | (2) | F: CTGGTGGTTTTGAAGCAAAGATT | *glyA* | (10) | F: TAATGTTCAGCCTAATTCAGGTTCTC |
| | | | R: CAATACCAGTGTCTAAAGTGCGTTTAT | | | R: GAAGAACTTACTTTTGCACCATGAGT |
| | | | **P: TXR-TTCCAACATCGCTAATGTATA-MGB-NFQ** | | | P: LC670-AATCAAAGCCGCATAAACACCTTGATTAGC-BBQ |
| *Campylobacter* spp. | 16S rRNA gene | (3) | F: CACGTGCTACAATGGCATAT | NP | NP | NP |
| | | | R: GGCTTCATGCTCTCGAGTT | | | NP |
| | | | P: FAM-CAGAGAACAATCCGAACTGGGACA-BHQ1 | | | NP |
| *Clostridium difficile* toxin A/B | *tcdB* | (4) | F: GAAAGTCCAAGTTTACGCTCAAT | *tcdB* | (4) | F: GAAAGTCCAAGTTTACGCTCAAT |
| | | | R: GCTGCACCTAAACTTACACCA | | | R: GCTGCACCTAAACTTACACCA |
| | | | P: FAM-ACAGATGCAGCCAAAGTTGTTGAATT-BHQ1 | | | P: YAK-ACAGATGCAGCCAAAGTTGTTGAATT-BBQ |
| *Cryptosporidium* spp. | 18S rRNA gene | (5) | F: ATGACGGGTAACGGGGAAT | DnaJ-like protein gene | (11) | F: CTTTTTACCAATCACAGAATCATCAGA |
| | | | R: CCAATTACAAAACCAAAAAGTCC | | | R: TGTGTTTGCCAATGCATATGAA |
| | | | P: TXR-CGCGCCTGCTGCCTTCCTTAGATG-BHQ2 | | | **P: LC670-TCGACTGGTATCCCTATAACT-BBQ** |
| *Cyclospora cayetanensis* | 18S rRNA gene | (6) | F: TAGTAACCGAACGGATCGCATT | 18S rRNA gene | (6) | F: TAGTAACCGAACGGATCGCATT |
| | | | R: AATGCCACGGTAGGCCAATA | | | R: AATGCCACGGTAGGCCAATA |

| | | | | | | |
|---|---|---|---|---|---|---|
| | | | P: FAM-CCGGCGATAGATCATTCAAGTTTCTGACC-BHQ1 | | | P: YAK-CCGGCGATAGATCATTCAAGTTTCTGACC-BBQ |
| *Entamoeba histolytica* | 18S rRNA gene | (7) | F: ATTGTCGTGGCATCCTAACTCA | 18S rRNA gene | (7) | F: ATTGTCGTGGCATCCTAACTCA |
| | | | R: GCGGACGGCTCATTATAACA | | | R: GCGGACGGCTCATTATAACA |
| | | | **P: YAK-TCATTGAATGAATTGGCCATTT-BHQ1**[B] | | | P: FAM-TCATTGAATGAATTGGCCATTT-BBQ |
| EIEC / *Shigella* | *ipaH* | NA[A] | F: GAATTTACGGACTGGTTCTCC | *ipaH* | (12) | F: CCTTTTCCGCGTTCCTTGA |
| | | | R: TCTCATATTTCTGCTCTTCTGC | | | R: CGGAATCCGGAGGTATTGC |
| | | | P: FAM-CCCAGCGGTCAGCTTCCGT-BHQ1 | | | P: FAM-CGCCTTTCCGATACCGTCTCTGCA-BBQ |
| *Giardia lamblia* | 18S rRNA gene | (7) | F: GACGGCTCAGGACAACGGTT | 18S rRNA gene | (7) | F: GACGGCTCAGGACAACGGTT |
| | | | R: TTGCCAGCGGTGTCCG | | | R: TTGCCAGCGGTGTCCG |
| | | | P: FAM-CCCGCGGCGGTCCCTGCTAG-BHQ1 | | | P: YAK-CCCGCGGCGGTCCCTGCTAG-BBQ |
| Norovirus GI | RdRp / Capsid junction | (1) | F: CGYTGGATGCGNTTYCATGA | RDRP / Capsid junction | (13,14) | F: GCYATGTTCCGCTGGATGC |
| | | | R: CCTTAGACGCCATCATCATTTAC | | | R: CGTCCTTAGACGCCATCATCA |
| | | | **P: YAK-TYGCGRTCTCCTGTCCA-BHQ1**[B] | | | P: FAM-ATTCGGGCAGGAGAT-MGB-BBQ |
| Norovirus GII | RdRp / Capsid junction | (1) | F: CARGARBCNATGTTYAGRTGGATGAG | RdRp / Capsid junction | (1) | F: CARGARBCNATGTTYAGRTGGATGAG |
| | | | R: TCGACGCCATCTTCATTCACA | | | R: TCGACGCCATCTTCATTCACA |
| | | | **P: YAK-AGATYGCGATCSCCCTC-BHQ1**[B] | | | **P: FAM-AGATYGCGATCSCCCTC-BBQ** |
| *Plesiomonas shigelloides* | *hugA* | NA[A] | F: CGGTAAAGTGGTCATAAC | NP | NP | NP |
| | | | R: CGGCGATATTTATGAAGATA | | | NP |
| | | | P: FAM-CACTGACTACCTAATGGAATATCGGC-BHQ1 | | | NP |
| Rotavirus | NSP3 | (1) | F1: ACCATCTTCACGTAACCCTC | NSP3 | (1) | F1: ACCATCTTCACGTAACCCTC |
| | | | F2: ACCATCTACACATGACCCTC | | | F2: ACCATCTACACATGACCCTC |
| | | | R: CACATAACGCCCCTATAGCC | | | R: CACATAACGCCCCTATAGCC |
| | | | P: TXR-ATGAGCACAATAGTTAAAAGCTAACACTGTCAA-BHQ2 | | | P: LC670-ATGAGCACAATAGTTAAAAGCTAACACTGTCAA-BBQ |
| *Salmonella* spp. | *ttrB* | NA[A] | F: ACCGTTACGCCATGCTTATC | *ttrBCA* | (15) | F: CTCACCAGGAGATTACAACATGG |
| | | | R: TTGGTTTTCAATAGTGCAACTTAC | | | R: AGCTCAGACCAAAAGTGACCATC |
| | | | P: YAK-TGACAGCCGATACAACGCCGCA-BHQ1 | | | P: YAK-CACCGACGGCGAGACCGACTTT-BBQ |
| Sapovirus (I, II, IV, V) | RdRP / | NA[A] | F1: AGGCTCTCGCCACCTA | RdRP / | (16) | F1: GAYCASGCTCTCGCYACCTAC |

| | | | | | | |
|---|---|---|---|---|---|---|
| | Capsid junction | | F2: GYTAYAACAGCTGGTACATWGG | Capsid junction | | F2: TTGGCCCTCGCCACCTAC |
| | | | R: RCCCTCCATYTCAAACACTA | | | F3: TTTGAACAAGCTGTGGCATGCTAC |
| | | | P1: ATTO700-TGYACCACCTATRAACCA-MGB-NFQ | | | R: CCCTCCATYTCAAACACTA |
| | | | P2: ATTO700-CAGAAATGCCCRCTACCAATGAA-BHQ3 | | | P1: FAM-CCRCCTATRAACCA-MGB-BBQ |
| | | | | | | P2: FAM-TGCCACCAATGTACCA-MGB-BBQ |
| STEC | *stx1* | (8) | F1: TGGCATTAATACTGAATTGTCATCATC | *stx1* | (17) | F: CATAGTGGAACCTCACGACGCAGT |
| | | | F2: TGGCATTAATATTAAATTGCCATCAT | | | R: TTTGCCGAAAACGTAAAGCTTCA |
| | | | **R1: GCGTAATCCCACGSACTCTT** | | | P: FAM-TGTGGCAAGAGCGATGTTACGGTTTG-BBQ |
| | | | R2: GAGTAATCCCACGCCCACTTC | | | |
| | | | P: **YAK-ATGAGTTTCCTTCTATG-MGB-NFQ** | | | |
| | *stx2* | (8) | F1: CCGGAATGCAAATCAGTCGT | *stx2* | (17) | F1: GTTTCCATGACAACGACAGCAG |
| | | | F2: GGAACGTACAGGGATGCAGATT | | | R1: GTGACGACTGATTTGCATTCCGG |
| | | | **R1: CCACTRAACTCCATTAACGCC** | | | R2: ACGCCCAATCTGCATCCCT |
| | | | R2: CGTCCTCTGAACTCCATTAAATCC | | | P1: YAK-CAACGTGTCGCAGCGCTGGAAC-BBQ |
| | | | **P1: FAM-ACTCACTGGTTTCATCATA-MGB-BHQ1** | | | P2: YAK-ACAGCGAATCGCAGATCTGGAAC-BBQ |
| | | | **P2: FAM-ATATGAACCAACCAGTGAATGACGCC-BHQ1** | | | |
| *Yersinia enterocolitica* | *gyrB* | NA[A] | F: GCAGGCACCATTGAAAGTGG | *ail* | (18) | F: ATGATAACTGGGGAGTAATAGGTTCG |
| | | | R: CTCACGCAGACGTTTTGCC | | | R: CCCAGTAATCCATAAAGGCTAACATAT |
| | | | P: TXR-CCAGAAACGCACGGTTGTCCC-BHQ2 | | | P: FAM-TCTATGGCAGTAATAAGTTTGGTCACGGTGATCT-BBQ |

Nucleotide sequences in bold represent modifications of the published nucleotide sequences.

[A]Unpublished LDT-protocol that will be made available by the authors upon request.

[B]XS-probe™ (Biolegio™)

LDT – laboratory-developed real-time PCR assay, LUMC – Leiden University Medical center, RUMC – Radboud University Medical Center, EIEC - Enteroinvasive *Escherichia coli,* STEC - Shiga-like toxin producing *E. coli*, NA – not available, NP – not performed, F – forward primer, R – reverse primer, P – probe, YAK – Yakima Yellow®, TXR – Texas Red®, BBQ – BlackBerry® Quencher, BHQ – Black Hole Quencher®, NFQ – non fluorescent quencher, MGB – minor groove binder.

## REFERENCES


1. van Maarseveen NM, Wessels E, de Brouwer CS, Vossen AC, Claas EC. 2010. Diagnosis of viral gastroenteritis by simultaneous detection of adenovirus group F, astrovirus, rotavirus group A, norovirus genogroups I and II, and sapovirus in two internally controlled multiplex real-time PCR assays. J Clin Virol 49:205-210.
2. Best EL, Powell EJ, Swift C, Grant KA, Frost JA. 2003. Applicability of a rapid duplex real-time PCR assay for speciation of *Campylobacter jejuni* and *Campylobacter coli* directly from culture plates. FEMS Microbiol Lett 229:237-241.
3. Lund M, Nordentoft S, Pedersen K, Madsen M. 2004. Detection of *Campylobacter* spp. in chicken fecal samples by real-time PCR. J Clin Microbiol 42:5125-5132.
4. Knetsch CW, Bakker D, de Boer RF, Sanders I, Hofs S, Kooistra-Smid AM, Corver J, Eastwood K, Wilcox MH, Kuijper EJ. 2011. Comparison of real-time PCR techniques to cytotoxigenic culture methods for diagnosing *Clostridium difficile* infection. J Clin Microbiol 49:227-231.
5. Jothikumar N, da Silva AJ, Moura I, Qvarnstrom Y, Hill VR. 2008. Detection and differentiation of Cryptosporidium hominis and Cryptosporidium parvum by dual TaqMan assays. J Med Microbiol 57:1099-1105.
6. Verweij JJ, Laeijendecker D, Brienen EA, van Lieshout L, Polderman AM. 2003. Detection of *Cyclospora cayetanensis* in travelers returning from the tropics and subtropics using microscopy and real-time PCR. Int J Med Microbiol 293:199-202.
7. Verweij JJ, Blangé RA, Templeton K, Schinkel J, Brienen EA, van Rooyen MA, van Lieshout L, Polderman AM. 2004. Simultaneous detection of *Entamoeba histolytica*, *Giardia lamblia*, and *Cryptosporidium parvum* in fecal samples by using multiplex real-time PCR. J Clin Microbiol 42:1220-1223.
8. de Boer RF, Ferdous M, Ott A, Scheper HR, Wisselink GJ, Heck ME, Rossen JW, Kooistra-Smid AM. 2015. Assessing the public health risk of Shiga toxin-producing *Escherichia coli* by use of a rapid diagnostic screening algorithm. J Clin Microbiol 53:1599-1598.
9. Haramoto E, Katayama H, Oguma K, Ohgaki S. 2007. Quantitative analysis of human enteric adenoviruses in aquatic environments. J Appl Microbiol 103:2153-2159.
10. Jensen AN, Andersen MT, Dalsgaard A, Baggesen DL, Nielsen EM. 2005. Development of real-time PCR and hybridization methods for detection and identification of thermophilic *Campylobacter* spp. in pig faecal samples. J Appl Microbiol 99:292-300.
11. Bruijnesteijn van Coppenraet LE, Wallinga JA, Ruijs GJ, Bruins MJ, Verweij JJ. 2009. Parasitological diagnosis combining an internally controlled real-time PCR assay for the detection of four protozoa in stool samples with a testing algorithm for microscopy. Clin Microbiol Infect 15:869-874.

12. Vu DT, Sethabutr O, Von Seidlein L, Tran VT, Do GC, Bui TC, Le HT, Lee H, Houng HS, Hale TL, Clemens JD, Mason C, Dang DT. 2004. Detection of *Shigella* by a PCR assay targeting the *ipaH* gene suggest increased prevalence of shigellosis in Nha Trang, Vietnam. J Clin Microbiol 42:2031-2035.
13. Hoehne M, Schreier E. 2006. Detection of norovirus genogroup I and II by multiplex real-time RT-PCR using a 3'-minor groove binder-DNA probe. BMC Infect Dis 6:69.
14. Dreier J, Strömer M, Mäde D, Burkhardt S, Kleesiek K. 2006. Enhanced reverse transcription-PCR assay for detection of norovirus genogroup I. J Clin Microbiol 44:2714-2720.
15. Malorny B, Paccassoni E, Fach P, Bunge C, Martin A, Helmuth R. 2004. Diagnostic real-time PCR for detection of *Salmonella* in food. Appl Environ Microbiol 70:7045-7052.
16. Oka T, Katayama K, Hansman GS, Kageyama T, Ogawa S, Wu FT, White PA, Takeda N. 2006. Detection of human sapovirus by real-time reverse transcription-polymerase chain reaction. J Med Virol 75:1347-1353.
17. Pavlovic M, Huber I, Skala H, Konrad R, Schmidt H, Sing A, Busch U. 2010. Development of a multiplex real-time polymerase chain reaction for simultaneous detection of enterohemorrhagic *Escherichia coli* and enteropathogenic *Escherichia coli* strains. Foodborne Pathog Dis 7:801-808.
18. Lambertz ST, Nilsson C, Hallanvuo S, Lindblad M. 2008. Real-time PCR method for detection of pathogenic *Yersinia enterocolitica* in food. Appl Environ Microbiol 74:6060-6067.
